# Wild-Type, but Not Mutant N296H, Human Tau Restores Aβ-Mediated Inhibition of LTP in *Tau*^−/−^ mice

**DOI:** 10.3389/fnins.2017.00201

**Published:** 2017-04-24

**Authors:** Mariana Vargas-Caballero, Franziska Denk, Heike J. Wobst, Emily Arch, Chrysia-Maria Pegasiou, Peter L. Oliver, Olivia A. Shipton, Ole Paulsen, Richard Wade-Martins

**Affiliations:** ^1^Biological Sciences and Institute for Life Sciences, University of SouthamptonSouthampton, UK; ^2^Department of Physiology, Anatomy and Genetics, University of OxfordOxford, UK; ^3^Wolfson Centre for Age-Related Diseases, King's College LondonLondon, UK; ^4^AstraZeneca-Tufts Lab for Basic and Translational Neuroscience, Tufts University School of MedicineBoston, MA, USA; ^5^Department of Physiology, Development and Neuroscience, University of CambridgeCambridge, UK

**Keywords:** Alzheimer's disease, amyloid beta, frontotemporal dementia, tau, MAPT, N296H

## Abstract

Microtubule associated protein tau (MAPT) is involved in the pathogenesis of Alzheimer's disease and many forms of frontotemporal dementia (FTD). We recently reported that Aβ-mediated inhibition of hippocampal long-term potentiation (LTP) in mice requires tau. Here, we asked whether expression of human *MAPT* can restore Aβ-mediated inhibition on a mouse *Tau*^−/−^ background and whether human tau with an FTD-causing mutation (N296H) can interfere with Aβ-mediated inhibition of LTP. We used transgenic mouse lines each expressing the full human *MAPT* locus using bacterial artificial chromosome technology. These lines expressed all six human tau protein isoforms on a *Tau*^−/−^ background. We found that the human wild-type *MAPT* H1 locus was able to restore Aβ_42_-mediated impairment of LTP. In contrast, Aβ_42_ did not reduce LTP in slices in two independently generated transgenic lines expressing tau protein with the mutation N296H associated with frontotemporal dementia (FTD). Basal phosphorylation of tau measured as the ratio of AT8/Tau5 immunoreactivity was significantly reduced in N296H mutant hippocampal slices. Our data show that human *MAPT* is able to restore Aβ_42_-mediated inhibition of LTP in *Tau*^−/−^ mice. These results provide further evidence that tau protein is central to Aβ-induced LTP impairment and provide a valuable tool for further analysis of the links between Aβ, human tau and impairment of synaptic function.

## Introduction

Multiple lines of evidence indicate an interaction between amyloid-beta (Aβ) and tau protein in Alzheimer's disease, and tau protein is required for the effect of Aβ in many experimental paradigms. Thus, cultured neurons from tau knockout (*Tau*^−/−^) mice are not susceptible to Aβ-induced synaptic damage and neurotoxicity (Rapoport et al., 2002; Nussbaum et al., 2012; Zempel et al., 2013) or to Aβ-induced defects in axonal transport (Vossel et al., 2011). Reducing tau levels prevents Aβ-induced cognitive deficits and premature mortality, reduces spontaneous and induced seizure activity, and prevents synaptic impairment in mutant APP-expressing mouse models (Roberson et al., 2007, 2011). Furthermore, Aβ plays a permissive role for the spread of tau pathology *in vivo* (Pooler et al., 2015). Similarly, tau oligomers produce an acute inhibition of hippocampal LTP and memory (Fá et al., 2016), and inhibition of the tau kinases CDK5 or GSK-3β reduces Aβ-induced neuronal cell death and malfunction (Llorens-Marítin et al., 2014) suggesting that abnormal tau mediates some of the effects of Aβ.

Previous reports consistently show that Aβ inhibits long-term potentiation (LTP) in the hippocampus (Ondrejcak et al., 2010), a widely accepted cellular model for learning and memory (Bliss and Collingridge, 1993), and we have previously shown that hippocampal slices from *Tau*^−/−^ animals are not susceptible to human Aβ_42_-mediated LTP impairment (Shipton et al., 2011). Furthermore, our work and that of others have shown that inhibition of GSK-3β prevents LTP impairment following exposure to Aβ and prevents Aβ-mediated increase in tau phosphorylation at disease-relevant AT8 epitopes (Jo et al., 2011; Shipton et al., 2011). To further analyse the interaction of Aβ_42_ and human tau and build upon our previous work, here we asked whether expressing the human tau protein in mice on a *Tau*^−/−^ background (Dawson et al., 2001) can restore the Aβ_42_-mediated inhibition of LTP. For this, we studied transgenic mice expressing one of two variants of the human tau protein: either a wild type form, or a mutant form carrying the disease-associated mutation, N296H, known to lead to frontotemporal dementia (FTD; Iseki et al., 2001). Both transgenes contain the complete *MAPT* genomic locus, which allows for the study of aberrant splicing, a phenomenon which has been observed in a variety of FTDs and tauopathies (Spillantini et al., 1998; Takanashi et al., 2002). The N296H mutation, in particular, is a splice site mutation that affects the isoform ratio detected in the insoluble fraction of tau fragments obtained from patient brains (Iseki et al., 2001) leading to an increase in tau isoforms harboring four microtubule-binding repeats (4R). Like many other familial FTD mutations (Denk and Wade-Martins, 2009), N296H causes increased inclusion of *MAPT* exon 10, leading to an overrepresentation of tau isoforms with four microtubule-binding repeats, known as 4R tau (Grover et al., 2002; Yoshida et al., 2002).

We hypothesized that the expression of wild type human tau would restore the inhibitory effect of Aβ_42_ on synaptic plasticity in *Tau*^−/−^ mice. Further, we tested whether mutant N296H tau would either occlude or prevent the Aβ_42_-mediated inhibition of hippocampal LTP. Elucidating the extent to which normal or mutant human tau protein can restore Aβ_42_-mediated inhibition of LTP in *Tau*^−/−^ mice should further enhance our understanding of the interaction between Aβ_42_ and tau protein in synapse dysfunction.

## Materials and methods

### Ethical statement

Animal care and experimental procedures were conducted in accordance with UK Home Office regulations under the Animals (Scientific Procedures) Act of 1986. The work was carried out under PPL 30/2757 and all efforts were made to optimize the number of animals used.

### Transgenic *MAPT* mice

Mice were generated using bacterial artificial chromosome (BAC) technology to express the 143 kb *MAPT* locus (Supplementary Methods) in mice with a genetic knock-out of endogenous tau (*Tau*^−/−^; Dawson et al., 2001) on a C57/BL6-J background. This allowed the physiological expression of all human tau isoforms. We chose to express the more common H1 haplotype given its association with increased risk of Alzheimer's disease. Mice expressed either *MAPT-H1* or the same transgene with the point mutation N296H. Two lines of N296H P1-derived artificial chromosome (PAC) transgenic mice (N24 and N51) were generated to control for insertion effects. Samples for analysis of tau isoform expression with Western blot were subjected to alkaline phosphatase treatment (Lambda protein phosphatase NEV, 4,000 U per 80 μg lysate) and assessed with the human specific Tau-13 antibody (1:5000 Abcam). Both male and female mice were used in each experiment.

### RNA *In situ* hybridization

*In situ* hybridization for transgene expression was carried out on frozen 14 μm thick tissue sections using a DIG-labeled LNA probe (Exiqon) designed to be specific for human *MAPT* (5′ DIG-gctcagccatcctggttcaaa-DIG 3′). Hybridisation and signal detection were carried out as previously described (Jefferson and Volpi, 2010).

### Exon 10 splicing measurements

Quantitative analysis of exon 10 splice ratios was obtained using the Sequenom MassARRAY Platform. Briefly, RT-PCR was performed to amplify exon 4 through to exon 11 of the *MAPT* gene. Sequenom analysis was performed by the Wellcome Trust Centre for Human Genetics, Oxford, to determine the relative amount of exon 10 inclusion vs. exclusion using mass-assisted laser desorption/ionization—time of flight (MALDI-TOF). Each MALDI-TOF assay was repeated eight times in both forward and reverse direction, and *N* = 2–3 mice were tested per group. A control construct expressing a known 1:1 ratio of 4R:3R tau was used for normalization.

### Slice preparation and pharmacology

Following the procedure described in Shipton et al. (2011), parasagittal hippocampal slices (400 μm) were prepared after decapitation under deep isoflurane-induced anesthesia. After dissection in ice-cold artificial CSF (ACSF) containing (in mM) 126 NaCl, 3 KCl, 1.25 NaH_2_PO_4_, 2 MgSO_4_, 2 CaCl_2_, 25 NaHCO_3_, 10 glucose, pH 7.2–7.4, bubbled with carbogen gas (95% O_2_, 5% CO_2_), slices were maintained at room temperature (22–25°C) in a submerged-style holding chamber for at least 1 h and after that incubated in ACSF with or without freshly prepared 220 nM human Aβ_42_ (also referred to as Aβ_1–42_ Tocris, UK) in a submerged chamber for 1–3 h (Shipton et al., 2011; Um et al., 2012). Perfusion with a half-concentration of the drug continued after slices were transferred to the interface chamber for fEPSP recordings under the assumption that this reduction to 110 nM would not cause a washout of the Aβ_42_ effect on plasticity. LTP was induced with high frequency stimulation using a single 1 s long 100 Hz induction protocol. Mice used for electrophysiology were 4–7 months old. The electrophysiology data were obtained from 18 H1 mice, 15 N51 mice, and 6 N24 mice, Number of observations *(N)* represents the number of slices subject to each pharmacological treatment. Input-output, paired pulse and LTP data were obtained from the same slices. Only slices with a stable baseline (drift <10%) were used for LTP induction. A sample of concentrated Aβ_42_ (44 μM) was processed in the same way as above and collected at the time of LTP measurement for protein stain analysis (Supplementary Methods) to verify the presence of oligomeric Aβ assemblies in the solution (Supplementary Figure 1).

### Data analysis

Data were analyzed with one- or two-way ANOVA with genotype and treatment as independent variables. Data are represented as means ± SEM. Unless otherwise stated, *post-hoc* comparisons were corrected using Dunnett's method with H1 ACSF data as control.

To establish whether significant levels of LTP were obtained within each experimental group we performed a paired Student's *t*-test comparing baseline synaptic strength with synaptic strength at 40 ± 2.5 min post-HFS per individual experimental condition.

### Western blot

For analysis of slices incubated with or without addition of 220 nM Aβ_42_, following 1 h recovery at room temperature in ACSF, slices from 6 to 7 month old mice (*N* = 6 mice) were incubated for 2 h in either Aβ_42_ or control solutions. Slices were then snap frozen and collected for western blot analysis (Additional information in Supplementary Methods). Slices from the same mouse and treatment were pooled together resulting in *N* = 1 per mouse per treatment. AT8 and Tau-5 immunoreactivity was first normalized to GADPH. Immunoreactivity for both H1 and N51 was normalized to baseline H1 levels.

## Results

Following our previous observation that early LTP in mice with a genetic knock out of tau protein (*Tau*^−/−^) is not affected by Aβ, we wanted to investigate whether transgenic expression of human tau (*MAPT*) genes would allow normal LTP on a *Tau*^−/−^ background in mice and permit LTP inhibition by Aβ_42_.

**Figure 1:**
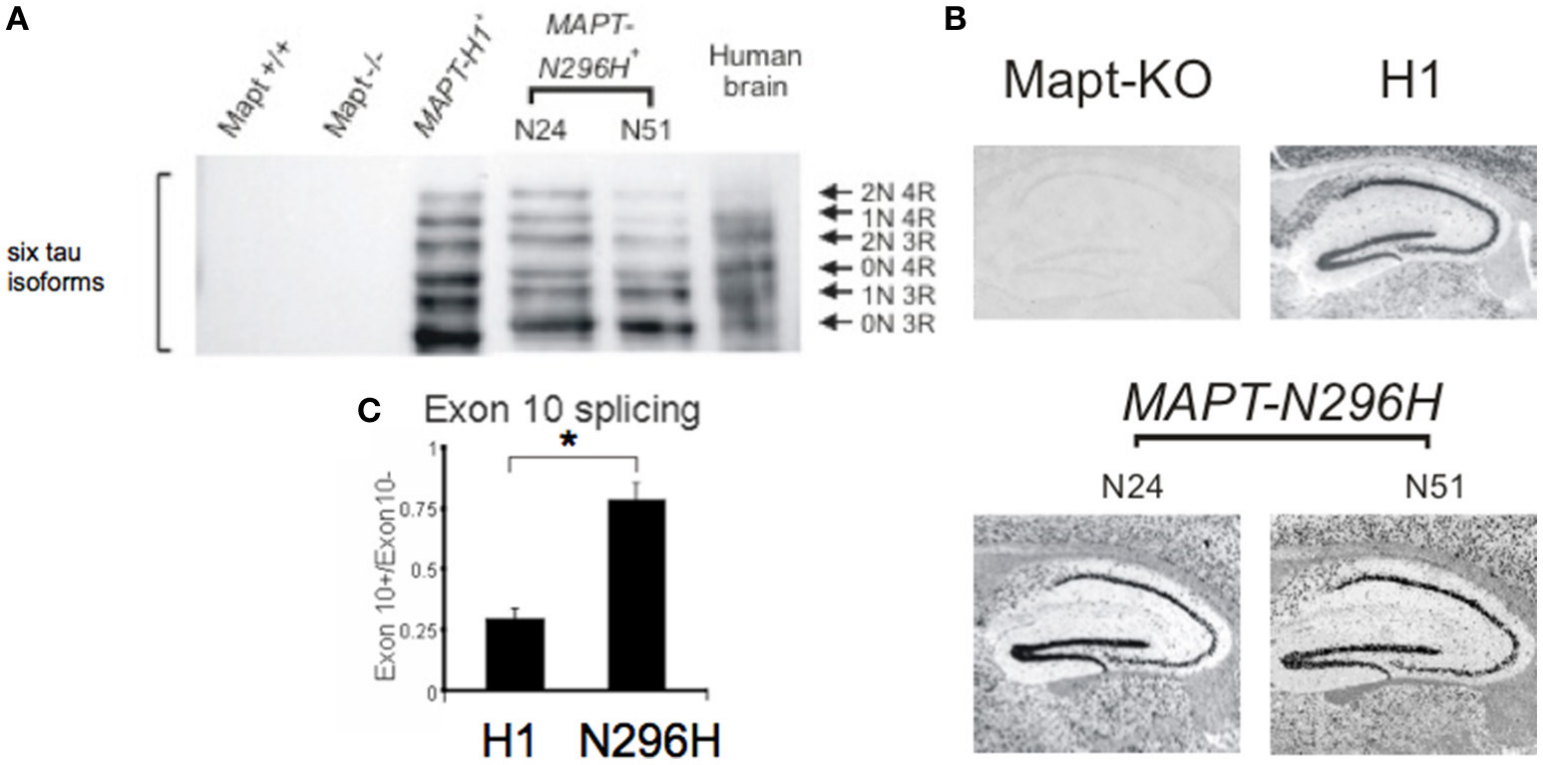
**Six isoforms of human tau protein in transgenic mouse lines ***MAPT-H1***, and ***MAPT-N296H*** expressed in a mouse ***Tau***^**−/−**^ (***Mapt***^**−/−**^) background. (A)** Western blot with a human-specific tau antibody. *MAPT-H1* and *MAPT-N296H* transgenic animals expressed all six isoforms of the human tau protein. **(B)** RNA *in situ* hybridization analyzing expression of *MAPT* in the hippocampus of *Tau*^−/−^ and *MAPT* mice. **(C)** Quantitative analysis of splice ratios of *MAPT* exon 10+(4R)/exon 10−(3R) RNA transcript in the brains from H1 and N296H (line N24) mice showing an enhanced ratio of exon 10 inclusion in N296H mice, ^*^p < 0.05.

We used three transgenic mouse lines. The first line carried the transgenic locus of wild-type human tau (line H1), and we used two independent lines carrying the tau N296H mutation linked to FTD with parkinsonism (lines N51 and N24). To identify the six tau isoforms we performed Western blot following a dephosphorylation treatment (Figure 1A) using a human tau specific antibody.

We wanted to test the effect of expressing tau protein on hippocampal LTP; to confirm that human tau mRNA was expressed in the hippocampus we used *in situ* hybridisation. We observed an enrichment of our signal in the hippocampal cell body layers (Figure 1B) in a pattern similar to that observed in the Allen brain atlas for adult mouse tau (Allen Institute for Brain Science, 2015). To test whether expression of normal or mutant human tau caused changes at the mRNA expression level we measured the ratio of exon10 inclusion vs. exon10 exclusion in mRNA transcripts and we found that *MAPT-N296H* had a significantly higher inclusion of exon 10 (Line H1 0.30 ± 0.005 *N* = 3, N296H—line 24—0.78 ± 0.02 *N* = 2, *P* < 0.05, Figure 1C).

To test synaptic function and LTP in hippocampal CA3-CA1 synapses we obtained acute brain slices from these transgenic mice. We incubated slices in either 220 nM oligomeric Aβ_42_ (Supplementary Figure 1) or control ACSF for 1–3 h.

**Figure 2:**
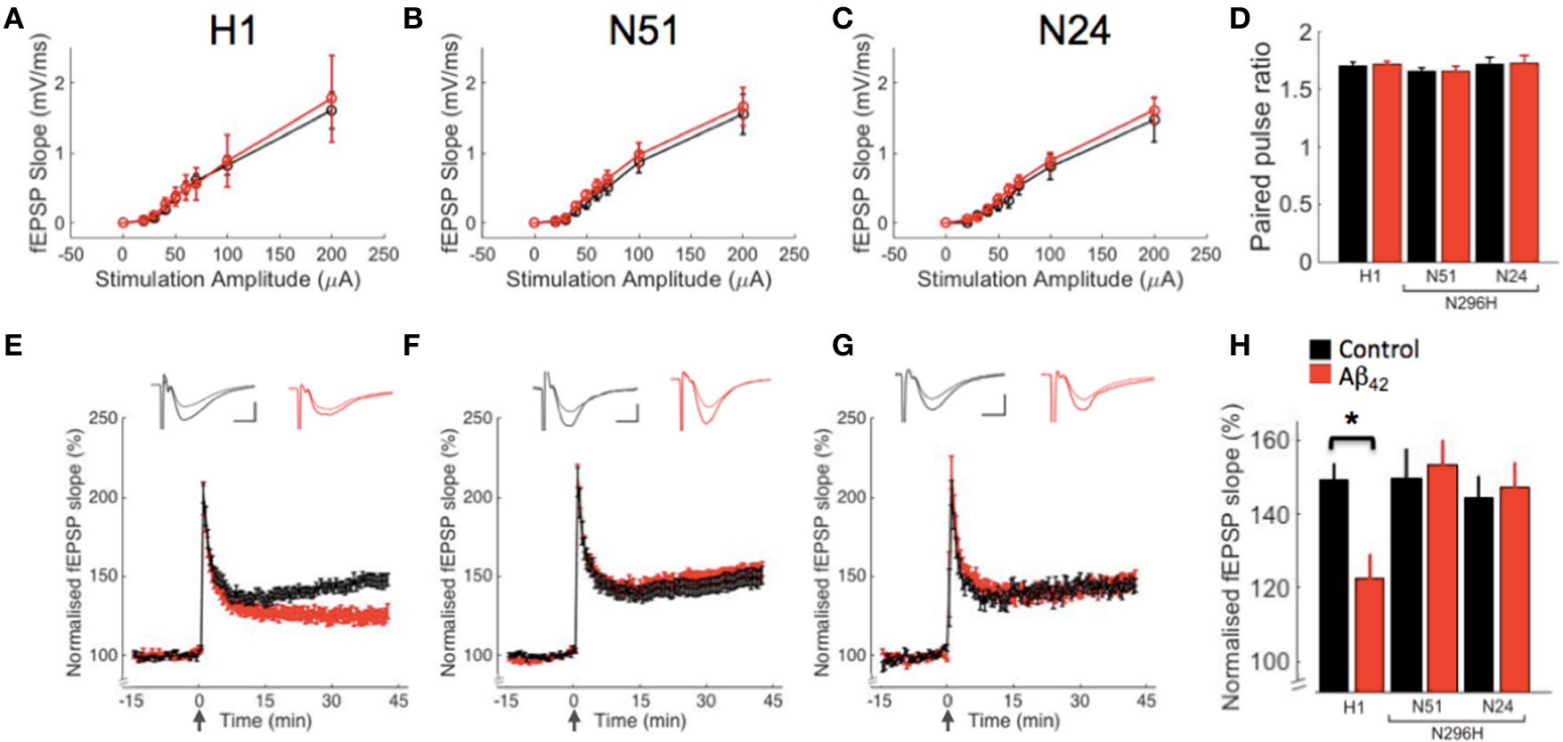
**Hippocampal LTP in slices from mice expressing human tau on a ***Tau***^**−/−**^ background is impaired by Aβ_**42**_, however Aβ_**42**_ does not reduce LTP in slices from mice expressing tau protein N296H associated with FTD**. Field recordings from CA3 to CA1 synapses in control ACSF (black) or after incubation with Aβ_42_ (red). **(A–C)** Synaptic input-output curves corresponding to genotypes above graph. **(D)** Paired-pulse data. **(E–G)** LTP in wild type human tau (H1) and two transgenic lines (N51, N24) expressing mutant tau (N296H). LTP was induced by delivering a high-frequency stimulation (HFS) train (100 Hz for 1 s). The insets show superimposed example traces before and 40 min after HFS for each condition. Scale bars: 5 ms/0.5 mV. **(H)** Summary of LTP results 40 min after HFS, ^*^P < 0.05.

We have previously shown that basal synaptic transmission is not affected following acute Aβ_42_ incubation in WT or *Tau*^−/−^ mice. To measure input-output relation, we evoked field excitatory postsynaptic potentials (fEPSPs) in CA1 with extracellular stimulation of Schaffer collaterals by delivering brief electrical pulses of increasing amplitude from 20 to 200 μA. We observed similar input-output curves for both ACSF and Aβ_42_ treatment in all three transgenic lines tested (Figures 2A–C). Repeated measures ANOVA between-subjects showed no significant effect of genotype [*F*_(2, 68)_ = 0.33, *P* = 0.72] or treatment [*F*_(1, 68)_ = 0.74, *P* = 0.39] and no genotype^*^treatment interaction [*F*_(2, 68)_ = 0.51, *P* = 0.49].

To characterize paired-pulse ratio as a measure of presynaptic function, we delivered paired stimuli 40 ms apart at CA3-CA1 synapses (Figure 2D). Two-way ANOVA on paired-pulse data showed no effect of genotype [*F*_(2, 82)_ = 1.67, *P* = 0.19] or treatment [*F*_(1, 82)_ = 0.06, *P* = 0.81] and no genotype^*^treatment interaction [*F*_(2, 82)_ = 0.02, *P* = 0.98].

LTP in the hippocampus is widely assumed to be a cellular correlate of learning and memory. We previously showed that 220 nM Aβ_42_ inhibits tetanus-induced LTP in wild type mice and that it does not have an effect on LTP in *Tau*^−/−^ mice (Shipton et al., 2011). We wanted to test whether transgenic expression of human tau restored the effect of Aβ_42_ on LTP in mice on a *Tau*^−/−^ background; for this we used mice with transgenic expression of tau in its wild type form (line H1) or with a point mutation related to FTD (N296H, lines N24 and N51). To analyse the effects of Aβ_42_ on LTP we monitored fEPSPs by stimulating Schaffer collaterals and induced LTP with tetanic (HFS) stimulation (Figures 2E–G).

We found significant LTP following HFS in H1 mice (148.43 ± 5.47, *N* = 16, *P* < 1 × 10^−8^ compared to baseline) and, interestingly, also normal LTP levels in both lines expressing tau with the N296H mutation (line N51 149.82 ± 7.40, *N* = 18, *P* < 1 × 10^−6^, line N24 144.32 ± 5.80, *N* = 7, *P* < 1 × 10^−5^). We hypothesized that in H1 mice, incubation of acute slices with Aβ_42_ peptide would significantly impair hippocampal LTP owing to the wild type human tau expression. Furthermore, we wanted to test the effects of human tau with the N296H mutation on LTP under control conditions and following Aβ_42_ incubation. Following from pilot experiments on WT slices demonstrating an inhibitory effect of Aβ_42_ on LTP as described in Shipton et al. (2011) (not shown), we interleaved control and Aβ_42_ incubation experiments and compared the resulting LTP for the three transgenic lines with and without Aβ_42_. Following a two-way ANOVA analysis of LTP results, we found an effect of genotype *F*_(2, 84)_ = 3.11, *P* < 0.05 on the induction of LTP and although no effect of treatment on LTP was observed *F*_(1, 84)_ = 0.82, *P* = 0.37, importantly a genotype^*^treatment interaction was found *F*_(2, 84)_ = 2.83, *P* < 0.05. fEPSP slopes in H1 slices with Aβ_42_ incubation were significantly different from baseline following HFS (LTP following Aβ_42_ incubation: 124.7 ± 6.2, *N* = 18, *P* < 1 × 10^−4^), however, *post-hoc* comparison between H1 in control conditions and H1 following incubation in Aβ_42_ revealed a significant difference between these treatments (*P* < 0.05, Figure 2H). Strikingly, there was no difference in LTP when comparing control and Aβ_42_ conditions in the N51 line with the N296H mutation (Aβ_42_ LTP, 153.6 ± 6.1, *N* = 20, *P* = 0.65 compared to control). These results showing no effect of Aβ_42_ on LTP in line N51 N296H mice are further supported by our data analyzing line N24, an independently generated line with the N296H mutation to control for transgene insertion effects. This line also showed normal LTP following Aβ_42_ incubation (Aβ_42_ LTP = 147.3 ± 6.3, *N* = 6, *P* = 0.50 compared to control levels, Figure 2H).

**Figure 3:**
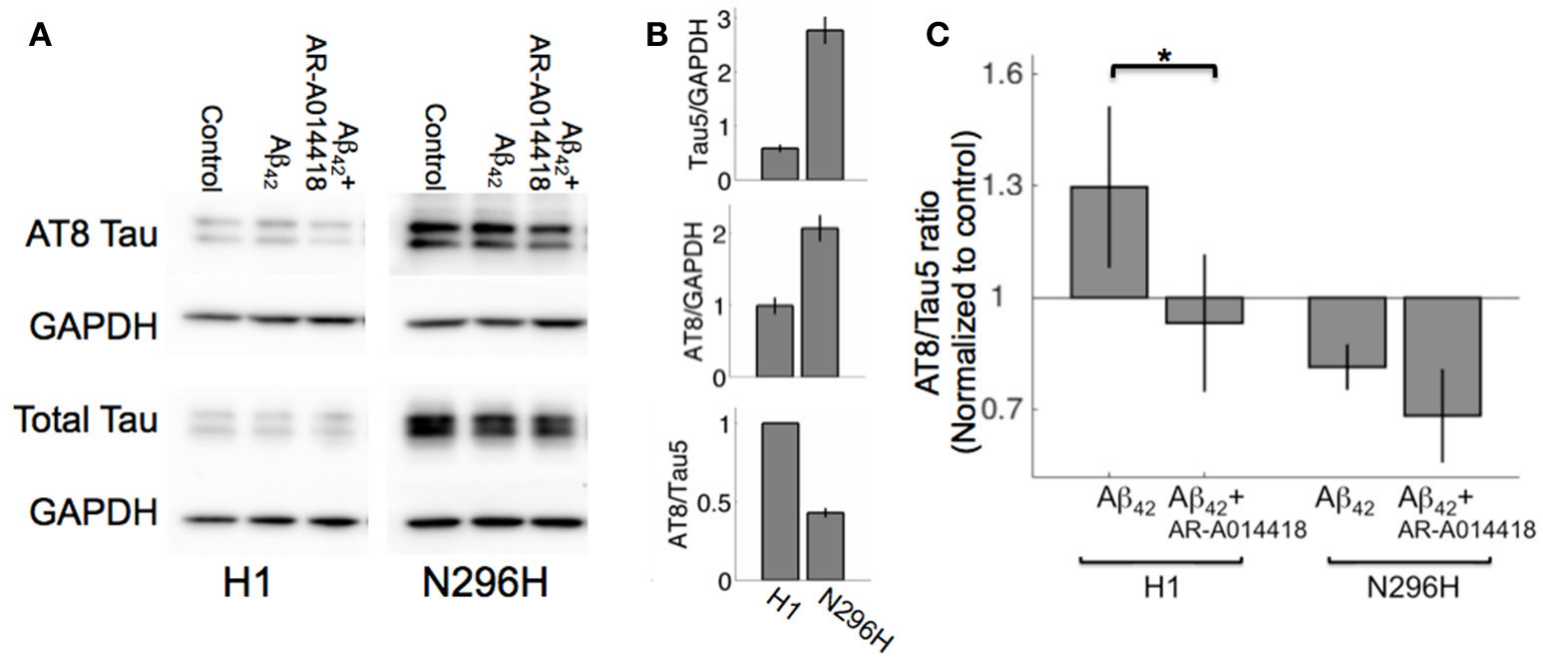
**Tau protein phosphorylation measured as AT8 to Tau5 immunoreactivity ratio is lower for N296H hippocampal slices compared to H1. (A)** Example Western blots showing immunoreactivity to AT8 (phosphorylated tau) and Tau5 (total tau) in slices from *MAPT-H1* and *MAPT-N256H* (line N51) mice incubated in control conditions, with Aβ_42_, or with Aβ_42_ + GSK3 inhibitor. **(B)** Quantification of phosphorylated (AT8) tau (top), total (Tau5) tau (middle) immunoreactivity relative to housekeeping protein GAPDH for both strains, and quantification of AT8/Tau5 immunoreactivity ratio (bottom) for both strains in basal conditions (untreated slices) for *N* = 6 mice each. **(C)** Quantification of AT8/Tau5 immunoreactivity ratio in control conditions and following Aβ_42_ or Aβ_42_ + GSK3 inhibitor incubation for *N* = 6 mice, each normalized to basal levels in each genotype ^*^P < 0.05.

In contrast to N296H mice, Aβ_42_ significantly inhibited LTP in H1 mice. We have previously shown that tau phosphorylation measured with AT8 immunoreactivity was increased following incubation with Aβ_42_ in hippocampal slices from WT mice (Shipton et al., 2011). In order to investigate whether an increase in tau phosphorylation is associated with Aβ_42_-mediated LTP impairment, we assessed total tau levels and tau phosphorylation levels in acute slices from H1 and N296H mouse hippocampal slices. We first noted that in control samples total tau levels normalized to GAPDH were 5.4 ± 0.3 times higher in N51 animals compared to H1 values normalized to GAPDH (*N* = 6 H1, *N* = 6 N51, *P* < 1 × 10^−5^. Figures 3A,B). However, phosphorylated tau measured with the AT8 antibody as a proportion of total tau measured with the Tau5 antibody with Western blot was significantly reduced in N51 hippocampal slices in control ACSF conditions compared to H1 mice (0.43 ± 0.03 of normalized values from H1 mice, *N* = 6 H1, *N* = 6 N51, *P* < 1 × 10^−5^, Figure 3B).

We wanted to test whether a significant increase in phosphorylation would be observed in either H1 or N51 mouse hippocampus following incubation with Aβ_42_ as we previously reported in WT mice, and whether this could be prevented by using the specific GSK3 inhibitor AR-A014418 (Bhat et al., 2003). For this we normalized AT8/Tau5 level following Aβ_42_ or Aβ_42_ + AR-A014418 incubation to basal AT8/Tau5 levels as an internal control for each genotype. Two way ANOVA revealed an effect of genotype [*F*_(1, 32)_ = 5.27, *P* < 0.05], however we did not see a main effect of treatment [*F*_(2, 32)_ = 2, *P* = 0.15]. *Post-hoc* comparison of AT8/Tau5 ratios in control conditions between H1 and N296H resulted in a significant difference [H1 1.3 ± 0.2 (*N* = 6), N51 0.8 ± 0.1 (*N* = 6), *P* < 0.05], however, neither H1 nor N51 AT8/Tau5 ratio following Aβ_42_ incubation was significantly higher than normalized control levels within the same genotype (H1 *P* = 0.10; N51 *P* = 0.96; Figure 3C).

## Discussion

We have shown that wild type human tau is able to restore Aβ_42_-mediated inhibition of LTP measured in mouse hippocampal slices on a mouse *Tau*^−/−^ background. However, brain slices from transgenic mice expressing a version of tau protein with a point mutation associated with familial FTD (N296H, line N51) showed normal LTP both under control conditions and following Aβ_42_ incubation. We also assessed a second independently-derived mouse line expressing all six tau isoforms of N296H mutant tau (line N24) corroborating a mutation-dependent resistance to Aβ_42_-induced inhibition of LTP. Our data suggest that the N296H mutant tau acts like a functional knockout in the pathways that link Aβ_42_ to LTP impairment, since N296H mice like *Tau*^−/−^ mice are protected from the effects of Aβ_42_ peptide on LTP.

The bacterial chromosome transgenic technology we used for generation of transgenic animals allows for anatomical expression patterns at physiological levels, however it is not possible to match exact level of protein expression in all transgenic lines. We measured protein expression levels in H1 and N51 mice, with H1 mice showing lower levels of tau protein. In all three lines we were able to observe the six tau isoforms produced by alternative splicing from the human tau locus. Wild type human tau in H1 mice was able to restore the Aβ_42_-mediated phenotype on a *Tau*^−/−^ background, indicating sufficient protein levels to link Aβ_42_ with synaptic plasticity dysfunction. In N296H mice we observed normal basal levels of synaptic transmission and normal LTP. If N296H tau protein caused a gain of toxic function, a stronger phenotype would be expected (Roberson et al., 2007) from higher expression levels in line N296H-N51. However, we did not observe an enhanced effect under basal or Aβ_42_ conditions in slices with the N296H mutation and we therefore argue that expression levels did not confound our data.

We assessed phosphorylation levels of tau protein with the AT8 antibody compared to total levels of tau with the Tau5 antibody in H1 and N51 mice. A comparison of H1 and N51 lines showed that at basal levels the ratio of AT8 to Tau5 immunoreactivity was much higher in H1 hippocampi, however, these levels were not associated with dysfunction, as previously reported in WT mice (Shipton et al., 2011; Morris et al., 2015). We also did not observe tau aggregates in the H1 or N296H lines following AT8 immunohistochemistry in slices from up to 21 month old mice (data not shown). This is in contrast to a previously reported mouse line expressing the H1 haplotype under a P1-derived artificial chromosome with high expression levels on a *Tau*^−/−^ background, which showed abnormal tau hyperphosphorylation from 3 months of age (Andorfer et al., 2003).

The AT8 phosphorylation levels, synaptic input-output curves, paired-pulse ratios, and hippocampal LTP data presented here are comparable to our previous data from wild type and *Tau*^−/−^ mice (Shipton et al., 2011). Following our previous published observations and the electrophysiological recordings presented here we hypothesized that AT8 immunoreactivity would increase in H1 hippocampal slices incubated with Aβ_42_ compared to control conditions associated with inhibition of LTP. However, owing to low protein expression levels in H1 mice we had high variability in our Western blot analysis. Although we observed a trend of increased phosphorylation levels in H1 slices following Aβ_42_ incubation consistent with our previous findings, the results did not reach significance. It may be necessary to express H1 in homozygosity to yield a stronger phenotype for molecular analyses of tau phosphorylation and downstream events.

N51 mice expressing N296H mutant human tau, despite showing higher expression levels than H1 mice, showed starkly decreased levels of tau phosphorylation and were not susceptible to an Aβ_42_-induced increase in tau phosphorylation at the AT8 site. However, while these findings are congruent with a loss of function via a reduction in basal and Aβ_42_-induced tau phosphorylation, it is possible that the mutation, either in addition to or independent of its effects on tau phosphorylation, could lead to other changes in tau, such as other post-translational modifications. These could play a distinct role in the Aβ_42_ effects on hippocampal LTP that we do not rule out here.

There is mounting evidence for tau as a downstream mediator of Aβ effects (Rapoport et al., 2002; Roberson et al., 2007, 2011; Shipton et al., 2011; Vossel et al., 2011), however, the mechanisms by which tau mediates neuronal Aβ_42_-mediated dysfunction remains poorly understood. There is strong evidence that small soluble oligomers formed by phosphorylated forms of tau are highly toxic, however, it is unclear whether their early effects are driven by alterations in the cytoskeleton structure (Cowan and Mudher, 2013), by promoting downstream pathological cascades (De Strooper and Karran, 2016) or a combination of these. One potential set of mechanisms whereby the absence of functional tau in hippocampal neurons (i.e., *Tau*^−/−^ or MAPT-N296H) could prevent the effect of amyloid beta on LTP is by modifying the cellular distribution of fyn kinase. Under normal conditions the physical tau/fyn association results in targeting of fyn kinase to postsynaptic sites (Ittner et al., 2010) leading to basal phosphorylation of synaptic NMDARs at Y1472 by fyn which is notably reduced in *Tau*^−/−^ mice (Ittner et al., 2010). Aβ_42_ drives tau phosphorylation at GSK3β epitopes (Jo et al., 2011; Shipton et al., 2011; Mondragon-Rodriguez et al., 2012) by a variety of proposed mechanisms in the Wnt signaling pathway (Purro et al., 2012; Vargas et al., 2014). This tau phosphorylation results in enhanced dendritic fyn localization coupled to a disruption of the PSD-95/NMDAR interaction (Mondragon-Rodriguez et al., 2012) and abnormal postsynaptic density ultrastructure (Purro et al., 2012). The relationship between Aβ_42_-mediated disruption in glutamate uptake (Li et al., 2011) and tau phosphorylation is unclear, however this dysregulation has been reported to occur on the same time scale as in our present experiments and is well-placed to feed-back into the mechanism of Aβ_42_-mediated synaptic dysregulation on the time scale of hours. This suggests that the mechanisms described above could provide the basis of acute Aβ_42_-mediated LTP dysfunction. Here we show that normal but not mutant MAPT-N296H can restore Aβ_42_-mediated LTP dysfunction in H1 but not N296H mice. Our Western blot analysis demonstrates that MAPT-N296H is hypophosphorylated under basal conditions and that its phosphorylation measured at the AT8 epitope does not change following Aβ_42_ incubation in contrast to the phosphorylation increase observed in MAPT-H1 hippocampal slices. This suggests that the uncoupling of Aβ_42_ effects on LTP by MAPT-N296H occurs at the level of tau phosphorylation which could feed directly into the formation of small soluble tau oligomers (Cowan et al., 2010). This strengthens the link between Aβ_42_ signaling, tau phosphorylation at GSK3 epitope (detected by AT8 immunoreactivity) and LTP impairment observed in WT but not *Tau*^−/−^ mice (Shipton et al., 2011). Nevertheless, our experiments do not address whether the MAPT-N296H mutation in tau affects its association with fyn or its subcellular localization.

The mechanisms by which FTD-related mutations lead to neuronal dysfunction and loss are not well understood. However, there is evidence that distinct tau gain or loss of function mutations or progranulin mutations (in the absence of tau mutations) can lead to FTD (Baker et al., 2006; De Silva et al., 2006). Our results indicate that the N296H mutation in MAPT leads to hypophosphorylation and to a loss of function in the mechanisms that link Aβ_42_ with impairment of LTP. Therefore, the mechanism that causes neuronal dysfunction in N296H mutant cells may differ from that leading to tau hyperphosphorylation and downstream mechanisms in AD. We suggest that the human tau transgenic models we present here—whereby wild type H1 human tau mediates Aβ_42_-inhibition of LTP and N296H does not—can be used to further explore Aβ_42_-tau interactions in disease.

## Author contributions

Scientific concept and experimental design: MVC, RW, and OP. Data analysis: MVC and HW. Creation of mouse lines and tau western blot: FD. *In situ* hybridisation: PO. LTP recording: MVC, EA, and OAS. Western blot analysis of AT8/Tau5 ratio: HW. Aβ protein stain: CP. Wrote the manuscript: MVC and HW. All authors read and approved the final manuscript.

### Conflict of interest statement

The authors declare that the research was conducted in the absence of any commercial or financial relationships that could be construed as a potential conflict of interest.

## Supplementary material

Supplementary Figure 1 **(A)** Protein stain of ACSF with Aβ_42_ (at a concentration of 44 μM to allow detection) following 2 h from preparation showing oligomeric composition.
