# Supplementary figures and images for: Wild-Type, but Not Mutant N296H, Human Tau Restores Aβ-Mediated Inhibition of LTP in Tau−/− mice
Source: Front Neurosci. 2017 Apr 24;11:201. doi: 10.3389/fnins.2017.00201 (PMC5401872; doi:10.3389/fnins.2017.00201)

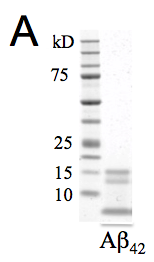

Supplement: Supplementary Figure 1 — (A) Protein stain of ACSF with Aβ42 (at a concentration of 44 μM to allow detection) following 2 h from preparation showing oligomeric composition. [file Image1.TIFF]
