# Supplementary material for: Wild-Type, but Not Mutant N296H, Human Tau Restores Aβ-Mediated Inhibition of LTP in Tau−/− mice
Source: Front Neurosci. 2017 Apr 24;11:201. doi: 10.3389/fnins.2017.00201 (PMC5401872; doi:10.3389/fnins.2017.00201)
Supplement: Supplementary file 2 [file DataSheet1.docx]

**Suplementary methods**

**Animals**

Animals had access to food and water ad libitum. Holding facilities were maintained at a temperature of 22°C, humidity of 60–70%, and with a 12 h light/dark cycle. We used animals of both sexes. MAPT-H1 line (Referred to as H1). For the generation of the H1 human tau line in a mouse tau knock out background (H1+Tau-/- also denoted as H1+Mapt-/-), in a C57/BL6 background male and female mice lacking microtubule associated protein tau (Tau−/−, Dawson et al, 2001) were maintained on a pure-bred C57BL6 background. The 143 kb wild-type human H1 MAPT locus from pBAC-MAPT26 originally from PAC61D06 (Genome Systems, St Louis, MO) was subcloned into the P1 bacteriophage-derived artificial chromosome (PAC) vector pCYPAC2 by homologous recombination gap-end joining using primers incorporating regions homologous to the MAPT genomic insert (lower case) and pCYPAC2 (CAPITALS): pCYPAC2_F (5’- tta agt gaa aat gta cag att gat tat ttt cac ctg gtt tct gtt aga tta tct tAA AAT CAT TTA ATT GGT GGT GCT GC - 3’) and pCYPAC2_R (5’- aga tag aaa ata tca tac agc tga ctt cac tag aga gaa agt gca tca act gct tAT TGA CCC GGA ACC CTT AAT ATA AC - 3’) to create pPAC-MAPT-H1. P1-artificial chromosomal (PAC) vectors containing a 143 kb MAPT transgene encoding either the wild-type MAPT locus or the N296H mutation engineered using BAC recombineering methods, were prepared by CsCl double banding for microinjection onto C57BL6 (MAPT-H1) or C57BL6/CBA (MAPT-N296H) mouse pronuclei. Founder pups were screened for the presence of the intact transgene by PCR and breeding lines were established.

Transgenic animals were backcrossed onto a pure C57BL6 Tau-/- background for a minimum of nine generations to obtain lines MAPT-H1+Tau-/- (referred to as the H1 line) and N296HTau-/- (referred to as the N24 or N51 lines). All transgenes were maintained as hemizygous on the homozygous Tau-/- background.

**Slice preparation.**

Parasagittal hippocampal slices (400 μm) were prepared after decapitation under deep isoflurane-induced anesthesia. After dissection in ice-cold artificial CSF (ACSF) containing (in mM) 126 NaCl, 3 KCl, 1.25 NaH2PO4, 2 MgSO4, 2 CaCl2, 25 NaHCO3, 10 glucose, pH 7.2–7.4, bubbled with carbogen gas (95% O2, 5% CO2), slices were maintained at room temperature (22-25°C) in a submerged-style holding chamber for at least 1 h and then incubated in drug/control solutions. For recording, slices were transferred to an interface-style recording chamber maintained at 33–35°C and superfused with ACSF at a rate of 2 ml/min, and recording started at least 15 min after the slices were transferred.

**Electrophysiological recordings.**

Data were acquired using MatDAQ (HPC Robinson) with an National Instruments Board Synaptic efficacy was monitored by stimulating the Schaffer collaterals (50 us, 20 – 60 uA) at 0.2 Hz (stimulation frequency was 0.1Hz in 3 out of 7 experiments with the N24 line) with a monopolar tungsten electrode connected to a stimulus isolator unit (ISO-Flex, A.M.P.I.). Stimulation strength was set to elicit a field EPSP (fEPSP) of half-maximal amplitude. fEPSP slopes were monitored for a baseline period of at least 15 min. If synaptic transmission was stable (< 10% change in fEPSP slopes over 15 min), a single high-frequency stimulus train was delivered (100 Hz for 1 s). To measure paired-pulse ratio both before and after LTP induction, two 50 us pulses with an interpulse interval of 40 ms were given at a low stimulation strength.

**Aβ characterization using Imperial Protein Stain**

hAβ42 was initially dissolved in ACSF to a concentration of 5uM. Aliquots were then sonicated for 11 min and incubated for 2hrs with 95%O2/5%CO2 bubbling.

At the end of incubation period a sample was loaded to a 4-12% Bis/Tris gel, charged to 70V for 10 minutes to get a good separation of the bands and then the voltage was increased to 120V and left to run for an hour.

After electrophoresis, the gels were placed in clean trays and washed 3 times for 5 minutes with ultrapure water with gentle shaking. The Protein Stain (24615/Thermo Scientific) was mixed thoroughly and 25 ml of stain were used to cover the gels and were left to incubate overnight with gentle shaking. Before imaging, the gels were washed with ultrapure water for at least 5 minutes and then imaged using the Odyssey® CLx Infrared Imaging System.

**Western blot** Two sections each from the same animal were treated with negative aCSF control or Abeta peptide, solutions. After treatment was completed the hippocampus in each slice was dissected out and snap-frozen in dry ice then stored at -80°C. Hippocampal slices were lysed in ice-cold radio-immunoprecipitation buffer (RIPA buffer: 50 mM Tris, 150 mM NaCl, 0.1% SDS, 0.5% sodium deoxycholate, 1% IGEPAL CA-630, pH 7.4) supplemented with protease inhibitors (cOmplete EDTA-free, Roche) and phosphatase inhibitors (PhosSTOP, Roche). Following homogenization, lysates were centrifuged for 5 min at 1 300 rcf and 4°C to remove cellular debris. Supernatants were collected and pellets discarded. Protein concentrations of lysed samples were determined by bicinchoninic acid (BCA) assay. Proteins in tissue lysates were reduced and denatured by boiling samples for 5 - 10 min in Laemmli buffer (60 mM Tris pH 6.8, 2% SDS, 10% glycerol, 5% β-mercaptoethanol, 0.01% bromophenol blue). Proteins (10 - 20 μg) were separated by SDS polyacrylamide gel electrophoresis (SDS-PAGE) and transferred onto polyvinylidene difluoride membranes (PVDF, Millipore). The following primary antibodies were used for Western blots: AT8 1:1 000, Tau-5 1:10 000 (Thermo Scientific), GAPDH 1:1 000 (Abcam). All antibodies were diluted in 5% skim milk/TBS-T. Secondary antibodies used were goat anti-rabbit and anti-mouse IgG-HRP conjugate (BioRad). ImageJ software (http://imagej.nih.gov/ij, National Institute of Health) was used for densitometric analysis of protein bands.
